# The endocannabinoidome mediator *N*-oleoylglycine is a novel protective agent against 1-methyl-4-phenyl-pyridinium-induced neurotoxicity

**DOI:** 10.3389/fnagi.2022.926634

**Published:** 2022-10-14

**Authors:** Anna Lauritano, Irene Cipollone, Roberta Verde, Hilal Kalkan, Claudia Moriello, Fabio Arturo Iannotti, Vincenzo Di Marzo, Fabiana Piscitelli

**Affiliations:** ^1^Endocannabinoid Research Group, Institute of Biomolecular Chemistry (ICB), National Research Council (CNR), Pozzuoli, NA, Italy; ^2^Institut Universitaire de Cardiologie et de Pneumologie de Québec, Université Laval, Québec City, QC, Canada; ^3^Institut sur la Nutrition et les Aliments Fonctionnels, Centre NUTRISS, Université Laval, Québec City, QC, Canada

**Keywords:** Parkinson, SH-SY5Y, endocannabinoidome, *N*-oleoylglycine, PPARα

## Abstract

*N*-oleoylglycine (OlGly) is a lipid mediator that belongs to the expanded version of the endocannabinoid (eCB) system, the endocannabinoidome (eCBome), which has recently gained increasing attention from the scientific community for its protective effects in a mouse model of mild traumatic brain injury. However, the effects of OlGly on cellular models of Parkinson’s disease (PD) have not yet been investigated, whilst other lipoaminoacids have been reported to have beneficial effects. Moreover, the protective effects of OlGly seem to be mediated by direct activation of proliferator-activated receptor alpha (PPARα), which has already been investigated as a therapeutic target for PD. Therefore, this study aims to investigate the possible protective effects of OlGly in an *in vitro* model obtained by treating the neuroblastoma cell line, SH-SY5Y (both differentiated and not) with 1-methyl-4-phenyl-pyridinium (MPP^+^), which mimics some cellular aspects of a PD-like phenotype, in the presence or absence of the PPARα antagonist, GW6471. Our data show that MPP^+^ increases mRNA levels of PPARα in both non differentiated and differentiated cells. Using assays to assess cell metabolic activity, cell proliferation, and pro-inflammatory markers, we observed that OlGly (1 nM), both as treatment (1 h) and pre-treatment (4 h), is able to protect against neuronal damage induced by 24 h MPP^+^ exposure through PPARα. Moreover, using a targeted lipidomics approach, we demonstrate that OlGly exerts its effects also through the modulation of the eCBome. Finally, treatment with OlGly was able also to reduce increased IL-1β induced by MPP^+^ in differentiated cells. In conclusion, our results suggest that OlGly could be a promising therapeutic agent for the treatment of MPP^+^-induced neurotoxicity.

## Introduction

Parkinson’s disease (PD) is an age-related progressive neurodegenerative disease characterized by the loss of nigrostriatal dopaminergic (DAergic) neurons and the accumulation of intracellular inclusions, known as Lewy bodies, which are composed primarily of alpha-synuclein in the substantia nigra pars compacta (SNpc). In PD, diminished striatal dopaminergic signaling triggers a cascade of neurochemical alterations, some of which are thought to account for the generation of motor symptoms, while others represent endogenous processes that attempt to compensate for the loss of dopamine (Rodriguez-Oroz et al., 2009).

The development of a stable and reliable DAergic neuronal cell model is particularly important to study the pathogenesis of PD and develop new therapeutic strategies. Increasing evidence suggests that the SH-SY5Y human neuroblastoma cell line is an ideal *in vitro* PD model because of its many features typical of DAergic neurons (Kovalevich and Langford, 2013). Many pharmacological or genetic approaches to reproduce a PD-like phenotype have been reported in the literature, and among these, the most-used compound in drug-based strategy is 1-methyl-4-phenyl-pyridinium (MPP^+^) (Huang et al., 2015), which specifically interferes with mitochondrial complex I activity and has been shown to induce mitochondrial dysfunction, by causing electron transport chain activity deficiency and thus increasing mitophagy and apoptosis in the substantia nigra (SN) (Vila and Przedborski, 2003). MPP^+^ is the active neurotoxic metabolite of 1-methyl-4-phenyl-1,2,3,6-tetrahydropyridine (MPTP), a by-product in the synthesis of 1-methyl-4-phenyl-4-propionoxy-piperidine (MPPP), a synthetic analog of heroin, which causes severe parkinsonism in humans when injected intravenously (Langston et al., 1983). Since SH-SY5Y cell lines do not have the enzyme monoamine oxidase-B (MAO-B) necessary to convert MPTP into MPP^+^ in the brain, this latter metabolite is directly administered, to induce neurotoxicity and to be used as an *in vitro* model for PD (Sheehan et al., 1997; Xicoy et al., 2017; Risiglione et al., 2020).

Although the etiology of PD is not completely understood, increasing evidence suggests that the endocannabinoid (eCB) system, a complex pleiotropic signaling system, might be a potential target for an effective therapeutic strategy in PD. The eCB system is traditionally described as composed of two cannabinoid-responsive G protein-coupled receptors (CB_1_ and CB_2_), their endogenous ligands, known as endocannabinoids (*N*-arachidonoylethanolamine or anandamide, AEA, and 2-arachidonoylglycerol, 2-AG), and five enzymes responsible for endocannabinoid biosynthesis and degradation (Iannotti and Piscitelli, 2018). Higher levels of CB_1_ receptors have been observed in the two key regions involved in movement control, such as the globus pallidus (GP) and the SN, besides other brain areas important for memory, cognition, and emotional manifestations (hippocampus, frontal-limbic, caudate putamen, cerebellum, and striatum) (Herkenham et al., 1990; Giuffrida and Martinez, 2017; Toczek and Malinowska, 2018). Moreover, the GP and SN exhibit also the highest concentrations of AEA (Di Marzo et al., 2000a,b). CB_2_ receptors are mainly expressed in the periphery and immune system, and although evidence showed that they are expressed in the CNS at lower concentrations than CB_1_, it seems that they are not involved in cortico-striato-pallidal circuit modulation (Behl et al., 2020). However, CB_2_ activation on astrocytes and microglia plays a key role in cytotoxicity and neuroinflammation (Piomelli, 2003; Giuffrida and Martinez, 2017). In particular, the components of the eCB system are highly expressed at different levels in the neural circuits of the basal ganglia, where they bidirectionally interact with dopaminergic, glutamatergic, and GABAergic signaling systems (Di Filippo et al., 2008). Within the motor areas of the brain, the endocannabinoid and dopamine systems regulate motor function and synaptic plasticity by modulating excitatory and inhibitory neurotransmission (Giuffrida and Martinez, 2017). Alterations of this cross-talk have been linked to the pathophysiology of PD and the maladaptive plasticity associated with the disabling motor complications caused by the long-term use of L-DOPA.

In particular, the eCB signaling system shows a biphasic pattern of changes during the progression of PD (Polissidis et al., 2013; Behl et al., 2020). Thus, while early and presymptomatic stages, characterized by neuronal malfunctioning, are associated with downregulation of CB_1_, advanced stages of parkinsonism, characterized by a profound nigral degeneration, are associated with upregulatory responses of CB_1_, and possibly CB_2_ too (García-Arencibia et al., 2009). Indeed, anatomical studies provided evidence for widespread distribution of CB_1_ in several regions, particularly in the striatum, by establishing a close functional interaction with dopaminergic neurotransmission and the glutamatergic system, supporting the involvement of this receptor in motor control (Di Filippo et al., 2008). Moreover, our group showed that 2-AG levels are enhanced in the GP of an animal model of the disease and that stimulation of dopamine receptors decreased both eCBs, AEA, and 2-AG (Di Marzo et al., 2000b). More recently, Celorrio et al. (2016) demonstrated the protective effects of URB597 (3′-Carbamoyl[1,1′-biphenyl]-3-yl cyclohexyl carbamate), a potent inhibitor of the main hydrolytic enzyme of AEA and other eCBs, the fatty acid amide hydrolase (FAAH), in MPTP-lesioned mice by inhibiting dopaminergic neuronal death and improving motor impairment. Moreover, the administration to SH-SY5Y cells of 4-Nitrophenyl 4-[di(2H-1,3-benzodioxol-5-yl)(hydroxy)methyl]piperidine-1-carboxylate (JZL184), a potent inhibitor of the main degradative enzyme of 2-AG, the monoacylglycerol lipase (MAGL), was found to exert neuroprotective effects (Aymerich et al., 2016).

In the last few decades, a huge number of naturally occurring *N*-acyl-amines, including other *N*-acylethanolamines (NAEs), *N*-acyl amino acids, and *N-*acyldopamine/taurine/serotonins, chemically related to the endocannabinoids and belonging to the complex lipid signaling system now known as endocannabinoidome (eCBome) (Iannotti and Piscitelli, 2018), have been discovered. Among *N*-acyl amino acids, *N*-oleoylglycine (OlGly), a member of the *N*-acylglycine family, has recently gained increasing attention from the scientific community for its efficacy in treating nicotine addiction and opiate withdrawal (Donvito et al., 2019; Petrie et al., 2019; Ayoub et al., 2021). These effects seem to be mediated by direct activation of proliferator-activated receptor alpha (PPARα) (Donvito et al., 2019). Moreover, recently we have demonstrated that OlGly ameliorates behavioral alterations in mice that underwent a mild traumatic brain injury (mTBI), while concomitantly modulating eCB and eCB-like mediator tone (Piscitelli et al., 2020).

Considering these previous data, this study aims to investigate the potential protective effects of OlGly against neurotoxicity induced by MPP^+^, as a model of neuronal injury in SH-SY5Y cells. In particular, we investigated:

1. The gene expression levels of molecular targets (CB_1_, CB_2_, and PPARα), biosynthetic (NAPE-PLD, DAGLα, and DAGLβ), and degradative (FAAH and MAGL) enzymes of eCBs and eCB-like mediators in not differentiated and differentiated SH-SY5Y cells, treated or not with MPP^+^ for 24 h;
2. The protective effects of OlGly treatment and pre-treatment, in the presence/absence of a PPARα antagonist GW6471, in the same cells (both differentiated and not) in delaying/arresting MPP^+^-induced neuronal injury, using assays to assess cell metabolic activity, cell proliferation, and pro-inflammatory markers;
3. The modulation of the eCBome in these cells after treatment and/or pre-treatment with OlGly, using a targeted lipidomics approach.

## Materials and methods

### Materials

The human neuroblastoma cell line, SH-SY5Y, was purchased from American Type Culture Collection (ATCC) (Manassas, VA, USA). Dulbecco’s Modified Eagle Medium (DMEM), fetal bovine serum (FBS), penicillin and streptomycin (P/S), non-essential amino acids, phosphate-buffered saline (PBS) pH 7.4, and Trypsin/EDTA solution were purchased from GIBCO (Grand Island, NY, USA); 1-methyl-4-phenylpyridinium iodide (MPP^+^) and dimethyl sulfoxide (DMSO) from Sigma-Aldrich (St. Louis, MO, USA); Retinoic Acid (RA) from Tocris Biosciences (Bethesda, MD, USA); GW 6471 and *N*-Oleoylglycine from Cayman Chemical Company (Ann Arbor, MI, USA); ApoTox-Glo Triplex Assay kit (G6321) from Promega Corporation (Fitchburg, WI, USA); Human interleukin-1 beta (IL-1β) and Human TNF-α ELISA kits from RayBiotech Life (Peachtree Corners, GA, USA).

### Cell culture and treatment

**FIGURE 1:**
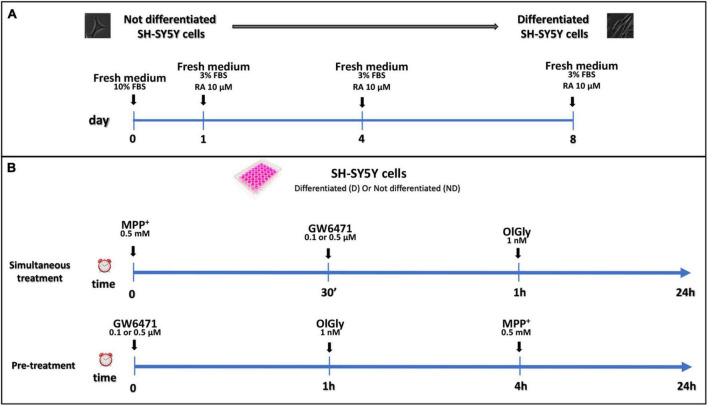
Differentiation protocol **(A)** and study design **(B)**. **(A)** Proliferative SH-SY5Y cells were seeded and cultured in a medium supplemented with 10% FBS for 24 h for complete adhesion. After adhesion, RA differentiation was induced with the reduction of FBS to 3% and the addition of 10 μM of RA. **(B)** Study design of simultaneous treatment (upper panel) and pre-treatment (bottom panel) experiments in not differentiated and differentiated SH-SY5Y cells. For simultaneous treatment experiments (**B**, upper panel), SH-SY5Y cells were exposed to MPP^+^ 0.5 mM to induce neurotoxicity. GW 6471 (0.5 or 0.1 μM) and OlGly (1 nM) were added 30 min and 1 h, respectively, after MPP^+^ treatment. For the pre-treatment experiments (**B**, bottom panel), prior to MPP^+^ treatment, SH-SY5Y was incubated with GW 6471 for 1 h and then with OlGly for 4 h.

Not differentiated SH-SY5Y cells were maintained in DMEM supplemented with 10% v/v inactivated FBS, 10,000 U/ml penicillin and 10 mg/ml streptomycin, and 1× non-essential amino acids; whereas differentiated SH-SY5Y cells were maintained in DMEM supplemented with a reduced percentage of inactivated FBS (3% v/v), and 10 μM RA for a minimum period of 8 days. Differentiated and not cells were stored at 37°C in a 95% humidified incubator with 5% CO_2_. The medium was changed every 4 days and the cells were used for no more than 25 passages. When the cells reached 80% confluence, they were detached using 0.2% (w/v) trypsin and transferred to different multi-wells according to the experimental procedure. Experiments were performed using 1.5 × 10^5^ cells/well into 24-well plates for MTT assay, 3 × 10^4^ cells/well into 96-well plates for ApoTox-Glo triplex assay, 4 × 10^5^ cells/well into 6-well cultured plates to targeted lipidomic approach to quantify eCBome mediators. In the simultaneous treatment experiment, 0.5 and 0.1 μM GW6471, and 1 nM OlGly compounds were added 30 min and 1 h, respectively, after the 0.5 mM MPP^+^ treatment. In the pre-treatment experiment, prior to MPP^+^ administration, cells were incubated with GW6471 for 1 h and then with OlGly for 4 h. The differentiation protocol and study design are reported in Figure 1.

### Cell viability assays

#### MTT and ApoTox-Glo triplex assay

Cell viability was measured by quantitative colorimetric assay with MTT, as described previously (Denizot and Lang, 1986). The medium was removed from each well of the plates; then, 300 μl of MTT reagent (0.5 mg/ml) was added and incubated in a humidified incubator at 37°C with 5% CO_2_ for 3 h period. Metabolically active cells convert the yellow MTT tetrazolium compound to a purple formazan product. The insoluble formazan was dissolved with 900 μl of isopropanol. The plates were placed on a shaker to solubilize the formations of purple crystal formazan. The absorbance was measured using a microplate reader at a wavelength of 570 nm. The results were used to construct a graph of cell viability percentage against control. Control cells treated with DMEM + Veh (Metanol/Ethanol < 0.03%) were taken as 100% viability.

The ApoTox-Glo triplex assay combines three Promega assay chemistries to assess viability, cytotoxicity, and caspase 3/7 activation events within a single assay well. After 24 h, 20 μl of viability/cytotoxicity reagent was added, containing both glycylphenylalanyl-aminofluorocoumarin (GF-AFC) and bis-alanyl-alanyl-phenylalanyl-rhodamine 100 (AAF-R110) substrates for cell viability and cytotoxicity measurements, respectively. Plates were placed on an orbital shaker set at 300 cycles for 30 s. Viable cells show a decrease in AFC fluorescence, while dead cells show an increase in R110 fluorescence. The mixture in a cover 96-well plate was incubated for 1 h at 37°C before the fluorescence signals were recorded with 400_*Ex*_/505_*Em*_ filters for viability and 485_*Ex*_/520_*Em*_ filters for cytotoxicity. Thereafter, caspase 3/7 activity was measured by adding 100 μl of the Caspase-Glo 3/7 reagent to all wells and the luminescence signal was measured after 30 min of incubation at room temperature. All signal measurements were performed using the GloMax Explorer Multimode Microplate Reader (Promega). Cell viability was expressed as a percentage of the control values.

### Western blot analysis

Total protein from SH-SY5Y cells was exacted using a 1× TNE buffer [50 mm Tris–HCl (pH 7.4); 100 mM NaCl. 0.1; mM EDTA) plus 1% (v/v) Triton X-100 (Cat# T8787, Sigma-Aldrich) and protease inhibitor (Cat# P8340, Sigma-Aldrich). Lysates were kept in an orbital shaker incubator at 220 rpm at 4°C for 30 min and then centrifuged for 15 min at 13,000 *g* at 4°C. The supernatants were transferred to tubes and quantified by DC Protein Assay (Cat# 5000116, Bio-Rad, Milan, Italy). Subsequently, protein samples (60 μg of total protein) were heated at 70°C for 10 min in 1× LDS Sample Buffer (Cat# B0007, Life Technology) plus 1× sample reducing agent (Cat# B0009, Life Technology) and loaded on 10% Bis-Tris Protein Gels (Cat# NW00102BOX, Life Technology) and then transferred the membrane using Trans-Blot Turbo Mini 0.2 μm PVDF Transfer Packs (Cat# 1704156 Bio-Rad). The primary antibodies used were: (a) rabbit anti-TH (Cat# NB300-109, Novus Biologicals) and (b) an anti-α-tubulin antibody (1D4) (Cat# T6199; Merk). Reactive bands were detected by Clarity Western ECL Substrate (Cat# 1705061 Bio-Rad). The intensity of bands was analyzed on a ChemiDoc station with Quantity-one software (Biorad, Milan, Italy).

### Targeted lipidomic approach to quantify endocannabinoidome mediators

About 24 h after completing the treatment, not differentiated and differentiated SH-SY5Y cells were collected and stored at −80°C until lipid extraction. Samples were then extracted with chloroform/methanol (2:1, v/v) containing internal deuterated standards for AEA, 2-AG, PEA, and OEA quantification by isotope dilution (5 pmol for [^2^H]_8_AEA; 50 pmol for [^2^H]_5_2-AG, [^2^H]_4_PEA, and [^2^H]_2_OEA). Homogenates were centrifuged at 2,000 rpm for 3 min and the organic phase was extracted four times with chloroform. Then the lipid extract was purified using open bed chromatography with silica gel. Fractions enriched in eCBs (9:1, CHCl_3_/CH_3_OH, v/v) were analyzed by liquid chromatography-atmospheric pressure chemical ionization-single quadrupole mass spectrometry S (LCMS-2020, Shimadzu, Milan, Italy), as previously described (Piscitelli et al., 2011). AEA, 2-AG, PEA, and OEA levels were calculated based on their area ratio with the internal deuterated standard signal areas.

### Interleukin-1 beta and tumor necrosis factor-α ELISA assays

Interleukin-1 beta and tumor necrosis factor-α (TNF-α) expression were measured using corresponding commercially available ELISA kits (Ray-biotech), according to the provided instructions.

### RNA extraction and quantitative PCR

Total RNA was isolated from SH-SY5Y cells by use of the TRIzol Reagent (Cat# 15596026; ThermoFisher, Italy), reacted with DNase-I (Cat# 180680151 U/μl; ThermoFisher, Italy) for 15 min at room temperature, followed by spectrophotometric quantification. The final preparation of RNA was considered DNA- and protein-free if the ratio between readings at 260/280 nm was ≥1.7. Isolated mRNA was reverse-transcribed by the use of iScript Reverse Transcription Supermix (Cat# 1708840; Biorad, Italy). Quantitative PCR (qPCR) was carried out in a real-time PCR system CFX384 (Bio-Rad) using the SYBR Green PCR Kit (Cat# 1725274, Biorad; Italy) detection technique and specific primer sequences reported in Supplementary Table 1. Each sample was amplified simultaneously in quadruplicate in a one-assay run with a nontemplate control blank for each primer pair to control for contamination or primer-dimer formation, and the cycle threshold (Ct) value for each experimental group was determined. The housekeeping gene ribosomal protein S16 was used to normalize the Ct values, using the 2^–Δ^ *^Ct^* formula. Differences in mRNAs content between groups were expressed as 2^–Δ^ ^Δ^ *^Ct^*, as previously described (Iannotti et al., 2018).

### Statistical analysis

Six replicates for each experimental condition were performed. Data are represented as mean values ± standard error of the mean (SEM). Comparisons between experimental and control among groups were performed by one-way ANOVA followed by Tukey’s *post hoc* test, using GraphPad Prism 9. Statistical difference was accepted when *p* < 0.05.

## Results

### 1-Methyl-4-phenyl-pyridinium induces an increase in proliferator-activated receptor alpha mRNA expression levels

**FIGURE 2:**
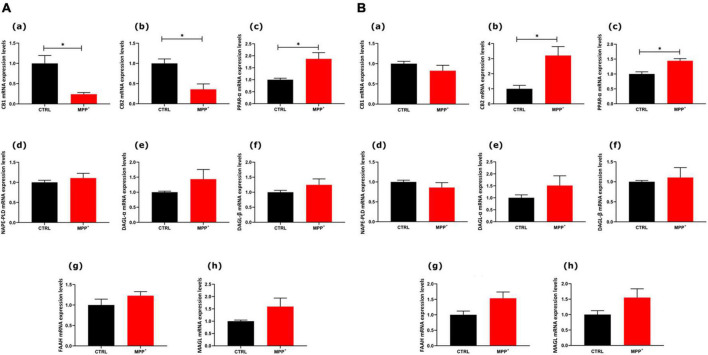
mRNA expression levels of indicated genes analyzed by quantitative PCR analysis. Differences in mRNAs content between groups were expressed as the 2^–ΔΔCt^ formula as reported in the material and method section in not differentiated **(A)** and differentiated **(B)** SH-SY5Y cells. Data are represented as mean ± SEM of at least three independent determinations. The asterisk (*) indicates a *p* < 0.05 vs. the control (veh) group.

To explore whether in SHSY5Y neuroblastoma cells the exposure to the neurotoxin MPP^+^ (24 h) could induce changes in the eCB system we analyzed mRNA expression of the entire set of genes encoding for their main receptors and metabolic enzymes of this system (Figure 2). In particular, the mRNA expression levels of the main receptors [CB_1_ (a), CB_2_ (b), and PPARα (c)] and enzymes [NAPE-PLD (d), DAGLα (e), DAGLβ (f), FAAH (g), and MAGL (h)] were measured in SH-SY5Y cells, both not differentiated (Figure 2A, ND) and differentiated (Figure 2B, D).

As shown in Figure 2A, in ND cells CB_1_ and CB_2_ decreased significantly in MPP^+^ treated cells, whereas PPARα mRNA expression levels increased (*p* < 0.05). Interestingly, in differentiated cells, PPARα mRNA still significantly increased after MPP^+^ and so did CB_2_ mRNA (*p* < 0.05). On the other hand, CB_1_ did not undergo any change in differentiated cells. The mRNAs encoding for enzymes, either biosynthetic or degradative, were not altered by MPP^+^.

### *N*-oleoylglycine treatment and pre-treatment increase cell viability in SH-SY5Y cells after 1-methyl-4-phenyl-pyridinium-induced neurotoxicity and reduce cytotoxicity

**FIGURE 3:**
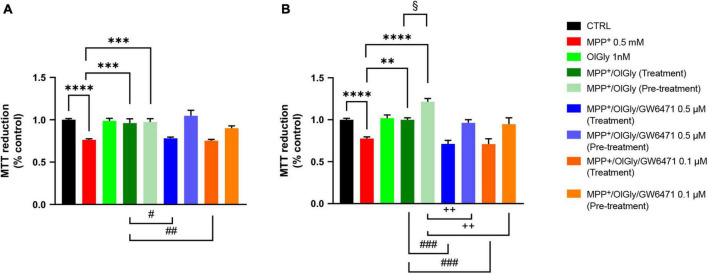
Cell viability in **(A)** not differentiated and **(B)** differentiated SH-SY5Y cells measured with the MTT assay. Histograms represent the percentage, with respect to control cells (CTRL, 100%), of viable cells after the exposure to MPP^+^ (0.5 mM), OlGly (1 nM), and GW 6471 (0.5 and 0.1 μM) in both simultaneous treatment and pre-treatment experiments. Data are represented as mean ± SEM. *Indicates values statistically significant vs. MPP^+^; ^#^ indicates values statistically significant vs. MPP^+^/OlGly (simultaneous treatment); ^+^ indicates values statistically significant vs. MPP^+^/OlGly (pre-treatment); ^§^ indicates values statistically significant of MPP^+^/OlGly (simultaneous treatment) vs. MPP^+^/OlGly (pre-treatment). Student’s *t*-test was used for statistical analysis (*N* = 6–12). ^****^Indicates values statistically significant with *p* < 0.0001; ^***^ and ^###^ indicates values statistically significant with *p* < 0.001; ^**^, ^##^, and ^ + +^ indicates values statistically significant with *p* < 0.01; *, ^#^, ^+^, and ^§^ indicates values statistically significant with *p* < 0.05.

We next moved on to examine whether changes in PPARα expression induced by MPP^+^ were associated with cell toxicity and if the effect was reverted by OlGly, which is known to act with a PPARα-mediated mechanism. Toward this goal, we exposed differentiated and not differentiated SHSY5Y cells to OlGly 1 nM, either as treatment (30 min) or pre-treatment (4 h), in the presence or absence of a selective PPARα receptor antagonist (GW6471, 0.5 and 0.1 μM). Cell viability measured using the MTT assay is reported in Figure 3. In particular, cell viability was measured as the percentage of cells that are able to reduce MTT and therefore are viable (expressed as % of control), in not differentiated (ND, Figure 3A) and differentiated (D, Figure 3B) SH-SY5Y cells. Previous pilot experiments were carried out to test the cytotoxicity of drugs (data not shown). In particular, we tested MPP^+^ at 1 and 0.5 mM, OlGly was tested at 2, 1, 0.1, 0.01, and 0.001 μM (EC_50_ 0.12 μM) and GW6471 was tested at 0.5, 0.1, 0.01, and 0.001 μM (IC_50_ 0.01 μM).

In both cell conditions, MPP^+^ (0.5 mM) reduced cell viability, by inducing about 25% mortality (*p* < 0.0001 vs. CTRL). OlGly at 1 nM had no effect on cell viability if administered alone but in combination with MPP^+^ was able to revert its effect by increasing cell viability to control levels (*p* < 0.001 and 0.01 vs. MPP^+^ in ND and D, respectively). However, in D cells, OlGly pre-treatment increased more significantly cell viability than simultaneous treatment (*p* < 0.05 vs. MPP^+^/OlGly). GW6471, a selective PPARα antagonist, was tested at two different concentrations (0.1–0.5 μM). As shown in Figure 3A, GW6471 was able to revert significantly the effect of OlGly treatment at both concentrations tested [*p* < 0.01 vs. MPP^+^/OlGly (treatment)]. On the other hand, GW6471 did not revert the OlGly effect on cell viability in the case of pre-treatment in ND cells. In D cells, GW6471 was still able to revert significantly the effect of OlGly treatment at both concentrations tested [###, p< 0.001 vs. MPP^+^/OlGly (treatment), for both concentrations]. GW6471 was able to revert also the effect of the pre-treatment with OlGly, although with less efficacy [*p* < 0.01 vs. MPP^+^/OlGly (pre-treatment) for both concentrations].

**FIGURE 4:**
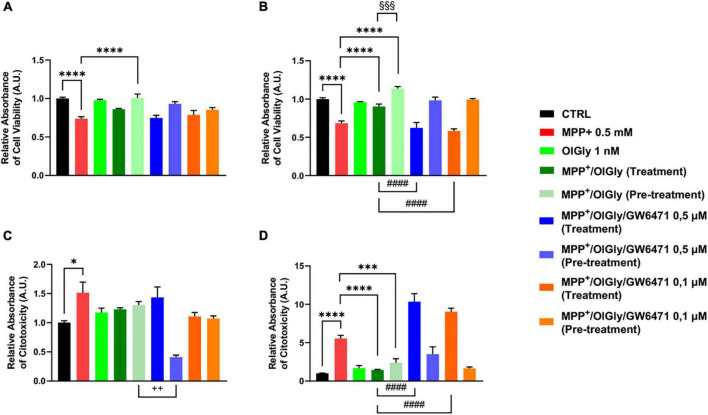
Cell viability **(A,B)** and cytotoxicity **(C,D)** in **(A,C)** not-differentiated and **(B,D)** differentiated SH-SY5Y cells measured with the ApoTox-Glo triplex assay. Histograms represent the percentage, with respect to control cells (CTRL, 100%), of viable cells **(A,B)** or dead cells **(C,D)** after the exposure to MPP^+^ (0.5 mM), OlGly (1 nM), and GW 6471 (0.5 and 0.1 μM) in both simultaneous treatment and pre-treatment experiments. Data are represented as mean ± SEM. *Indicates values statistically significant vs. MPP^+^; ^#^ indicates values statistically significant vs. MPP^+^/ OlGly (simultaneous treatment); ^+^ indicates values statistically significant vs. MPP^+^/ OlGly (pre-treatment); ^§^ indicates values statistically significant of MPP^+^/ OlGly (simultaneous treatment) vs. MPP^+^/ OlGly (pre-treatment). Student’s t-test was used for statistical analysis (*N* = 6). ^****^Indicates values statistically significant with *p* < 0.0001; ^***^, ^###^, and ^§§§^ indicates values statistically significant with *p* < 0.001; ^####^ indicates values statistically significant with *p* < 0.0001; ^ + +^ indicates values statistically significant with *p* < 0.01; * indicates values statistically significant with *p* < 0.05.

To confirm data obtained with the MTT assay, we next measured cell viability also with the ApoTox-Glo triplex assay, which is able to provide simultaneous results on cell viability, cytotoxicity, and activated caspase^3/7^ levels. As shown in Figure 4A, for ND cells, MPP^+^ reduced cell viability (*p* < 0.0001 vs. CTRL) and only the pre-treatment with OlGly 1 nM was able to increase viability (*p* < 0.0001 vs. MPP^+^). On the other hand, MPP^+^ increased cytotoxicity significantly (*p* < 0.05 vs. CTRL), while both treatment and pre-treatment with OlGly decreased cytotoxicity noticeably, although not reaching statistical significance (Figure 4C). However, both doses of GW6471 in the pre-treatment protocol decreased significantly cytotoxicity in comparison to the MPP^+^/OlGly group only at the highest concentration tested (*p* < 0.01). Regarding D cells, as shown in Figure 4B, the trend was the same as for the MTT assay. For instance, OlGly increased cell viability reduced by MPP^+^ (*p* < 0.0001 vs. CTRL), with both pre-treatment and simultaneous treatment [*p* < 0.0001 vs. MPP^+^ and *p* < 0.001 MPP^+^/OlGly (pre-treatment) vs. MPP^+^/OlGly (simultaneous treatment)]. Both doses of GW6471 reverted only the effect of the simultaneous treatment with OlGly (*p* < 0.0001 vs. MPP^+^/OlGly). Cytotoxicity was increased very significantly by MPP^+^, as shown in Figure 4D (*p* < 0.0001 vs. CTRL) and both treatment and pre-treatment with OlGly decreased it in a statistically significant manner (*p* < 0.0001 and *p* < 0.001 vs. MPP^+^, respectively). Both doses of GW6471 reversed the effect of OlGly significantly only with treatment (*p* < 0.0001 for both doses).

### *N*-oleoylglycine affects caspase^3/7^ activity in differentiated SH-SY5Y cells

**FIGURE 5:**
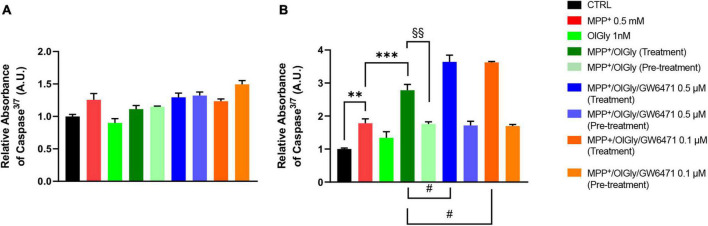
Levels of Caspase 3/7 activity in **(A)** not differentiated and **(B)** differentiated SH-SY5Y cells determined by using the ApoTox-Glo triplex assay. Histograms represent the percentage, with respect to control cells (CTRL, 100%), of viable cells after the exposure to MPP^+^ (0.5 mM), OlGly (1 nM), and GW 6471 (0.5 and 0.1 μM) in both simultaneous treatment and pre-treatment experiments. Data are represented as mean ± SEM. *Indicates values statistically significant vs. MPP^+^; ^#^ indicates values statistically significant vs. MPP^+^/ OlGly (simultaneous treatment); ^+^ indicates values statistically significant vs. MPP^+^/ OlGly (pre-treatment); ^§^ indicates values statistically significant of MPP^+^/ OlGly (simultaneous treatment) vs. MPP^+^/ OlGly (pre-treatment). Student’s *t*-test was used for statistical analysis (*N* = 6). ^***^Indicates values statistically significant with *p* < 0.001; ^**^ and ^§§^ indicates values statistically significant with *p* < 0.01; ^#^ indicates values statistically significant with *p* < 0.05.

1-Methyl-4-phenyl-pyridinium increased significantly caspase^3/7^ levels only in D (*p* < 0.01 vs. CTRL) cells, as shown in Figure 5 (Figure 5A, for ND cells and Figure 5B, for D cells). Neither OlGly simultaneous treatment nor pre-treatment did affect significantly caspase^3/7^ levels in ND cells (Figure 5A). On the other hand, in differentiated cells, OlGly treatment significantly increased caspase^3/7^ levels in comparison to MPP^+^ (*p* < 0.001) as well as both doses of GW6471 as compared to MPP^+^/OlGly (*p* < 0.05, for the two doses Figure 5B). Interestingly, pre-treatment with OlGly in D cells did not affect activated caspase^3/7^ in comparison to MPP+, but the effect was significantly reduced if compared with that of the simultaneous treatment [*p* < 0.01 vs. MPP+/OlGly (treatment), Figure 5B].

### *N*-oleoylglycine attenuated 1-methyl-4-phenyl-pyridinium-induced loss of tyrosine hydroxylase expression in differentiated SH-SY5Y cells

**FIGURE 6:**
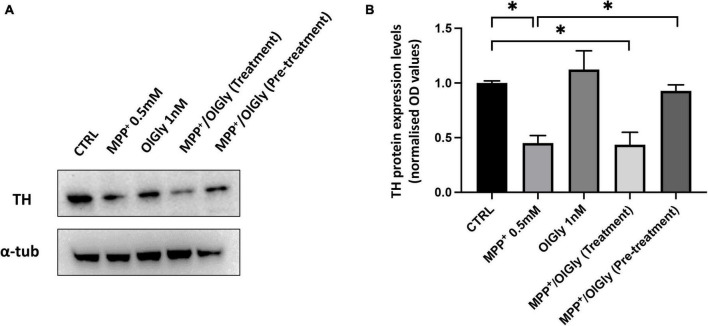
Expression of TH in differentiated SH-SY5Y cells. **(A)** Western blots of SH-SY5Y cell proteins with or without treatment with MPP^+^, OlGly, MPP^+^/OlGly (simultaneous treatment), and MPP^+^/OlGly (pre-treatment) probed with anti-TH-specific antibody. **(B)** Quantification of the data shown in **(A)**. The bars represent the ratio of anti-TH vs. α-tubulin expression. Data are expressed after normalization to the ratio OD TH/OD-tubulin in each group. Data are represented as mean ± SEM (*n* = 3). One-way ANOVA has been used for statistical analysis. Asterisks denote values statistically significant (*p* < 0.05).

Tyrosine hydroxylase (TH) is the rate-limiting enzyme in DA biosynthesis and the reduction of TH expression results in diminished DA synthesis leading to PD. Thus, TH plays a key role in the pathogenesis of PD. In this study, TH levels were measured using Western blotting. As shown in Figure 6, TH expression was decreased in MPP^+^-induced SH-SY5Y cells compared with control cells. OlGly (1 nM) alone did not have any effect on TH expression. In cells co-treated with MPP^+^, pre-treatment with OlGly effectively recovered TH expression, whereas the simultaneous treatment was ineffective. Thus, we suggest that OlGly pre-treatment is the most effective at attenuating MPP^+^-induced loss of TH expression in differentiated SH-SY5Y cells.

### *N*-oleoylglycine modulates endocannabinoid tone in both not differentiated and differentiated cells

**FIGURE 7:**
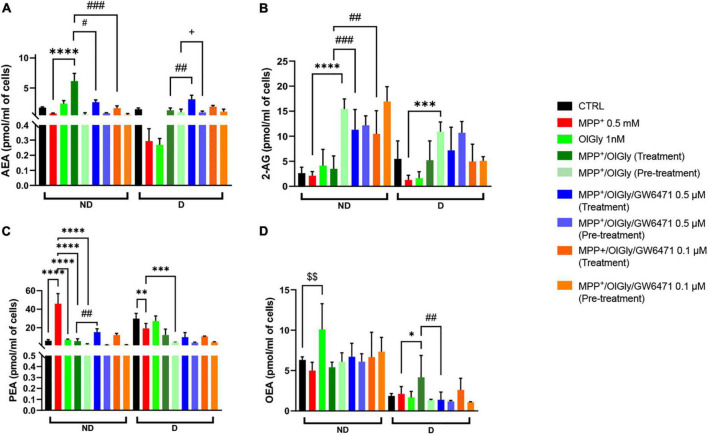
Levels of endocannabinoids (eCBs) and related *N*-acylethanolamines is not differentiated and differentiated SH-SY5Y cells. **(A)** Levels of anandamide (AEA) expressed as pmol/ml of cell culture medium. **(B)** Levels of 2-arachidonoyl glycerol (2-AG) expressed as pmol/ml of cell culture medium. **(C)** Levels of palmitoylethanolamide (PEA) expressed as pmol/ml of cell culture medium. **(D)** Levels of oleoylethanolamide (OEA) expressed as pmol/ml of cell culture medium. Data are represented as mean ± SEM. ^§^ indicates values statistically significant vs. control; *indicates values statistically significant vs. MPP^+^; ^#^ indicates values statistically significant vs. MPP^+^/OlGly (treatment). Student’s *t*-test was used for statistical analysis (*N* = 6–9). ^****^Indicates values statistically significant with *p* < 0.0001; ^***^, ^###^, and ^ + ⁣ + +^ indicates values statistically significant with *p* < 0.001; ^**^, ^###^, and ^§§^ indicates values statistically significant with *p* < 0.01; *, ^#^, and ^+^ indicates values statistically significant with *p* < 0.05.

Next, we moved on to explore whether OlGly may promote cell viability and neuroprotection also by modulating eCB tone. As shown in Figure 7, AEA (Figure 7A), 2-AG (Figure 7B), palmitoylethanolamide (PEA, Figure 7C), and oleoylethanolamide (OEA, Figure 7D) levels were measured in ND and D cells after OlGly simultaneous treatment and pre-treatment. AEA levels decreased after neurotoxicity induced by MPP^+^ in both ND and D (Figure 7A) cells, although not reaching statistical significance. Treatment with OlGly alone had no significant effect in both ND and D cells. Interestingly, OlGly in combination with MPP^+^ (treatment), only in ND cells, elevated AEA tone in a statistically significant manner as compared to MPP^+^ (*p* < 0.0001 Figure 7A). In ND cells both doses of GW6471 reverted the effect of OlGly treatment on AEA levels (*p* < 0.05 and 0.001 vs. MPP^+^/OlGly for 0.5 and 0.1 μM, respectively, Figure 6A). On the other hand, the highest dose of GW6471 increased AEA levels in D cells in comparison to MPP^+^/OlGly simultaneous treatment and pre-treatment groups (*p* < 0.01 and 0.05, respectively, Figure 7A).

2-Arachidonoylglycerol levels in ND were not affected by 1-methyl-4-phenyl-pyridinium and OlGly treatment, but both doses of PPARα antagonist increased 2-AG significantly as compared to MPP^+^/OlGly (*p* < 0.001 and 0.01 for 0.5 and 0.1 μM, respectively, Figure 7B). On the other hand, OlGly pre-treatment increased very significantly 2-AG levels in comparison to MPP^+^ (*p* < 0.0001, Figure 7B). As in ND cells, in D cells MPP^+^ had no significant effects on 2-AG levels in comparison to control (Figure 7B). However, OlGly pre-treatment, elevated the levels of 2-AG (*p* < 0.001 vs. MPP^+^, Figure 7B). No significant effect was observed with GW6471 (Figure 7B).

Interestingly, PEA increased almost ten times after MPP^+^-induced neurotoxicity (*p* < 0.0001 vs. CTRL, Figure 7C) in ND cells, whereas in D cells it decreased (*p* < 0.01, Figure 7C). In the MPP^+^/OlGly group, PEA levels instead decreased in both ND (*p* < 0.0001 vs. MPP^+^, with both simultaneous treatment and pre-treatment, Figure 7C) and D cells (*p* < 0.001 vs. MPP^+^, for pre-treatment, Figure 7C). GW6471 had significant effects only in not differentiated cultures by elevating PEA levels in comparison to MPP^+^/OlGly (*p* < 0.01 for 0.5 μM, Figure 7C).

Oleoylethanolamide levels in ND cells were not reduced significantly by MPP^+^ (Figure 7D), but were increased significantly by OlGly alone (*p* < 0.01 vs. CTRL, Figure 7D). Also in D cells MPP^+^ did not induce any significant effect on this mediator, whereas MPP^+^/OlGly (treatment) increased OEA levels (*p* < 0.05 vs. MPP^+^, Figure 7D) and the highest dose of GW6471 reduced them (*p* < 0.01 vs. MPP^+^/OlGly, Figure 7D). Pre-treatment did not induce any significant alteration of OEA levels (Figure 7D).

### *N*-oleoylglycine effect on pro-inflammatory markers: Interleukin-1 beta and tumor necrosis factor-α

**FIGURE 8:**
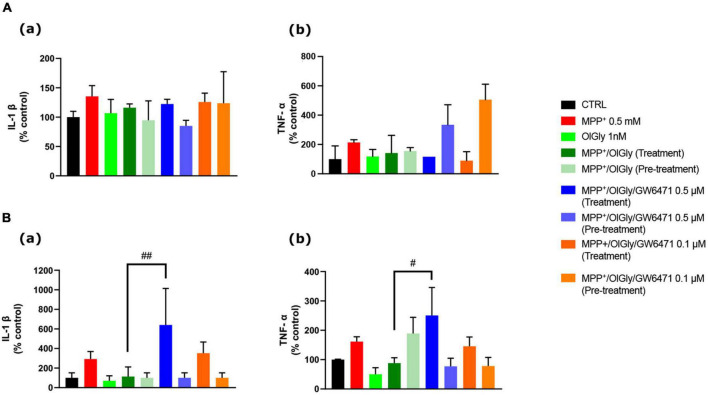
Effect of MPP^+^ on (a) Interleukin-1β (IL-1β) and (b) tumor necrosis factor-α (TNF-α) protein expression levels in **(A)** not differentiated and **(B)** differentiated SH-SY5Y cells measured by ELISA. Histograms represent the percentage, with respect to control cells (CTRL, 100%), of viable cells after the exposure to MPP^+^ (0.5 mM), OlGly (1 nM), and GW 6471 (0.5 and 0.1 μM) in both simultaneous treatment and pre-treatment experiments. Data are represented as mean ± SEM. *Indicates values statistically significant vs. MPP^+^. Student’s *t*-test has been used for statistical analysis (*N* = 6). ^##^Indicates values statistically significant with *p* < 0.01; ^#^Indicates values statistically significant with *p* < 0.05.

Finally, since it is well known that PD is accompanied by an inflammatory phenotype, we investigated the ability of OlGly to reduce the levels of two pro-inflammatory markers, IL-1β and TNF-α. In particular, Figure 8 reports the levels of these markers, IL-1β (a) and TNF-α (b), in ND (Figure 8A) and D (Figure 8B) cells. In both ND and D cells, MPP^+^ increased IL-1β levels although not reaching statistical significance. Both OlGly treatment and pre-treatment did not reduce significantly IL-1β levels, but GW6471 at the highest dose tested increased in a significant manner the levels of this interleukin [*p* < 0.01 vs. MPP^+^/OlGly (treatment)]. As for IL-1β, MPP^+^ did not induce an increase in TNF-α in either ND or D cells. Generally, TNF-α seemed not to be affected by simultaneous treatment and/or pre-treatment with OlGly, although in D cells it increased significantly with GW6471 at the highest dose tested [*p* < 0.05 vs. MPP^+^/OlGly (treatment)].

## Discussion

The present study described the protective effects of OlGly in human neuroblastoma SH-SY5Y cells incubated with the neurotoxin MPP^+^, an *in vitro* model of neurotoxicity which mimics some cellular aspects of a PD-like phenotype.

The human neuroblastoma cell line here used showed a fully functional eCB system in terms of receptors, enzymes, and endogenous ligands (Pasquariello et al., 2009; Aymerich et al., 2016; Lyons et al., 2020). Moreover, this cell line expresses also PPARα receptors (Avci et al., 2013), which have been suggested to be a promising therapeutic target in PD and other neurodegenerative diseases by their ability to counteract neuroinflammation (Barbiero et al., 2014; Zhang et al., 2015; Lee et al., 2019; Behl et al., 2021). To our knowledge, this is the first study in which the protective effect of OlGly on MPP^+^ treated cells in both not differentiated and differentiated neuronal cultures is reported, with a systematic investigation of cell viability, activated caspase^3/7^, pro-inflammatory markers, gene expression of biosynthetic and metabolic enzymes of eCBs, cannabinoid receptors, PPARα, and the endogenous levels of eCBs and related molecules. In fact, most of the studies present in the literature that have investigated the possible neuroprotective role of molecules targeting the eCB system, such as the MAGL inhibitor, JZL184 in the work of Aymerich et al. (2016) or Δ^9^-tetrahydrocannabinol (Zeissler et al., 2016), used only not differentiated cells in the former case and only differentiated cells in the latter case. Indeed, the suitability of differentiated or not differentiated SH-SY5Y cells as an effective *in vitro* model to investigate PD-induced neurotoxicity is still controversial. Differentiation with RA induces a general neuronal differentiation program leading to a predominantly mature DAergic-like neurotransmitter phenotype and increased expression of dopamine and noradrenalin transporters that are responsible for the uptake of MPP^+^ (Lopes et al., 2010; Luchtman and Song, 2010; Korecka et al., 2013). However, other studies reported that not differentiated cells are more susceptible to MPP^+^, while RA-differentiation confers to SH-SY5Y cells higher tolerance, potentially by up-regulating survival signaling pathways, suggesting that the real toxicity cannot be revealed in differentiated cells and the use of not differentiated SH-SY5Y is more appropriate to investigate neurotoxicity and/or neuroprotection in experimental PD research (Tieu et al., 1999; Cheung et al., 2009). For these reasons, in the present study we have fully investigated the neuroprotective effect of OlGly (simultaneous treatment and pre-treatment) in both not differentiated and RA-differentiated cells with the protocol shown in Figure 1.

Interestingly, our data show that the gene expression levels of cannabinoid receptors, CB_1_ and CB_2_, are quite different in not differentiated and differentiated cells following the toxicity induced by the MPP^+^. Indeed, while in ND cells both CB_1_ and CB_2_ mRNA levels decreased, following differentiation CB_1_ did not change and CB_2_ expression increased significantly, as compared to their control. However, comparing ND and D cells, the differentiation induced a noticeable increase in CB_1_ expression after MPP^+^. These results are not in agreement with the previous report of mRNA levels of both cannabinoid receptors in ND cells being higher in MPP^+^ treated cells (Aymerich et al., 2016). However, these authors used a higher concentration of MPP^+^ (5 mM) to induce a neurotoxic effect between 20 and 40% of mortality, whereas we report that a lower concentration (0.5 mM) of the neurotoxin is still effective, as elsewhere reported (Khwanraj et al., 2015), in both not differentiated and differentiated cells. In an elegant study, Korecka et al. (2013) carried out extensive genome wide transcriptional profiling combined with gene ontology, transcription factor, and molecular pathway analysis in RA-differentiated SHSY5Y cells demonstrating that RA induces a general neuronal differentiation program in SH-SY5Y cells as well as a mature dopaminergic phenotype. Moreover, this phenotype is characterized by increased dopamine levels and expression of dopamine and noradrenalin neurotransmitter transporters that are responsible for the uptake of MPP^+^. The increased neurotoxicity in RA-differentiated cells could provide an explanation for the differences observed between our results and those of Aymerich’s. In fact, the enhanced uptake of MPP^+^ induced by RA-differentiation increased the neurotoxicity of MPP^+^, as in Aymerich’s study, in which a higher concentration of neurotoxin was used, and also upregulated CB_1_ receptor expression, as compared to ND cells. However, the increase was not statistically significant if compared to the control in D cells.

On the other hand, PPARα mRNA expression levels significantly increased in both non differentiated and differentiated after MPP^+^ exposure, in agreement with the protective role of this receptor, as recently reviewed (Behl et al., 2021). Moreover, the role of PPARs in PD is well established. In particular, oral administration of the PPARγ agonist pioglitazone attenuated MPTP-induced glial activation and prevented dopaminergic cell loss in the SNpc, while PPARα activation by fenofibrate prevented the death of dopaminergic neurons of the SNpc in the MPTP model of PD, whereas bezafibrate, another PPARα agonist, was inactive (Dehmer et al., 2004). Moreover, Esposito et al. (2012) demonstrated that the neuroprotective effect of PEA was partially dependent on PPARα and the genetic ablation of this receptor in mice exacerbated MPTP systemic toxicity. Therefore, since our previous data showed that OlGly has a protective role in a mouse model of mTBI (Piscitelli et al., 2020) and is able to act through PPARα (Donvito et al., 2019), we decided to investigate the effect of this eCBome mediator in our *in vitro* model of MPP^+^-induced neurotoxicity. Moreover, oxidative stress, activation of the apoptotic cascade, and neuroinflammation have been confirmed to play central roles in the pathogenesis of PD (Maiti et al., 2017). The data presented here show that OlGly produces beneficial effects by restoring cell viability, in both MTT and ApoTox-Glo triplex assays, after MPP^+^ exposure, and decreased cytotoxicity and that in differentiated cells the effect is generally stronger than in not differentiated ones, with a more significant effect with pre-treatment than with the simultaneous treatment. Moreover, these effects were reverted with a selective antagonist of PPARα, demonstrating that OlGly acts through a PPARα-mediated mechanism, whereas in the case of pre-treatment the pro-survival effects were only partially reverted. On the other hand, we found that OlGly was not able to reduce caspase-dependent apoptosis induced by the neurotoxin, and in fact, in differentiated cells, OlGly treatment and especially the combination with GW6471 increased, even more, the levels of activated caspase^3/7^. In other studies, in which the role of PPARα was investigated in cancer, GW6471 (a PPARα selective antagonist) reduced tumor cell viability, interfering with the cell cycle and inducing apoptosis (Florio et al., 2017). In addition, Abu Aboud et al. (2013) demonstrated that GW6471 induced apoptotic death and cell cycle arrest and synergizes with glycolysis inhibition in renal cancer cells. Therefore, since SH-SY5Y cells are a neuroblastoma line, GW6471 might also induce programmed cell death by elevating the levels of caspases and OlGly might act on off-target(s). However, pre-treatment with OlGly did not affect caspase levels, and in fact, produced a significant decrease when compared to treatment, suggesting that this may be considered a safer protocol of administration in future experiments. Further investigation to confirm this hypothesis is required. The differences between pre-treatment and treatment showed interesting results that allow us to speculate about other possible mechanisms of action. In fact, controversial results in the literature exist about the effect of glycine on dopamine levels. Some studies reported an increase of serotonin, but not dopamine, levels in the prefrontal cortex of rats receiving an oral administration of glycine (Bannai et al., 2011), as well as the release of acetylcholine from superfused rat striatum, but not dopamine or glutamate, after stimulation with glycine (Hernandes et al., 2007). On the other hand, other studies reported the effectiveness of glycine at stimulating dopamine release (Javitt et al., 2005; Olsson et al., 2021). Interestingly, a recent study reported that OlGly is a positive allosteric modulator of glycine receptors (GlyR). In particular, the authors screened a plethora of *N*-acyl amino acids and found that C18 ω9 glycine increases both the potency of glycine activation of GlyRα_1_ and the maximal current generated by glycine (Gallagher et al., 2020). It is well known that stimulation of GlyR induces dopamine release *in vivo* (Yadid et al., 1993; Molander and Söderpalm, 2005; Höifödt Lidö et al., 2011). Therefore, it is tempting to hypothesize that our protocol of treatment with OlGly, but not pre-treatment, may induce the release of dopamine in differentiated cells through GlyRs, which in turn may induce activation of reactive oxygen species-dependent apoptosis, as previously reported (Sango et al., 2022).

We investigated the expression of tyrosine hydroxylase (TH), the enzyme required for catecholamine synthesis, in D cells as an indicator of functional differentiation. In fact, it has been reported that TH is constitutively expressed at very low levels in SH-SY5Y neuroblastoma cells and significantly increases after 3-day treatment with 10 μM RA (Kume et al., 2008). Moreover, another study reported a gradual decrease in TH in undifferentiated cells and a gradual increase in differentiated cells from days 4 to 10 after cell plating (Khwanraj et al., 2015). For this reason, we analyzed TH expression by western blot only in D cells after 7 days of differentiation. Accordingly, to what was already reported in the literature, in this study, MPP^+^ induced a significant decrease in TH expression (Figure 6), whereas OlGly alone had no effect. OlGly treatment and pre-treatment in combination with MPP^+^ induced different effects. In fact, only the pre-treatment was able to enhance significantly TH expression in comparison to MPP^+^. Thus, we suggest that OlGly pre-treatment attenuates MPP^+^-induced loss of TH expression in SH-SY5Y cells.

As already mentioned, in our previous work, in which OlGly protective effects were tested *in vivo* in the mouse model of mTBI, we demonstrated that this mediator was able to modulate eCB tone (Piscitelli et al., 2020). Also here, our data show that eCBs are altered by MPP^+^ and that OlGly restores the impaired eCB signaling. Generally, both AEA and 2-AG tended to decrease following MPP^+^, both in not differentiated and differentiated cells, and the combination of OlGly with the neurotoxin restored to control levels of the main eCBs, especially with the pre-treatment in the case of 2-AG. These data suggest that OlGly may affect eCB signaling, either by an *entourage* effect, as proposed for other eCB-like mediators (e.g., PEA) (Ho et al., 2008; Petrosino and Di Marzo, 2017), or by acting as a FAAH inhibitor (Donvito et al., 2019). PEA levels increased strongly in ND cells after MPP^+^, in agreement with its anti-inflammatory role (Petrosino and Di Marzo, 2017). On the other hand, in D cells, PEA levels were altered more similarly to AEA and 2-AG. In any case, OlGly was able to modulate also PEA levels following simultaneous treatment and pre-treatment. Interestingly, OlGly was able to module also OEA levels, even though only in D cells and in a way less effective than the other molecules analyzed. These effects are potentially important in as much as PEA and, particularly, OEA, are more potent PPARα agonists than OlGly (Fu et al., 2005; LoVerme et al., 2005), whose effect could thus also be due to changes in the levels of this mediators and not just to its interaction with the nuclear receptor.

Finally, to test the hypothesis that OlGly produces protective effects in this experimental model of MPP^+^-induced neurotoxicity also by counteracting neuroinflammation, the levels of two pro-inflammatory cytokines, IL-1β and TNF-α, were analyzed. Contrary to previous reports (Song et al., 2018; Ling et al., 2021), we found that 24 h treatment with MPP^+^ was not able to induce a significant increase of IL-1β and TNF-α, although we observed that it showed a tendency to increase. However, these authors used a higher concentration of MPP^+^ (2 and 1 mM, respectively) which could explain the difference in the data obtained. OlGly was not able to reduce significantly the levels of these cytokines, even though, there was a strong trend toward reduction. Only the highest concentration of GW6471, in the simultaneous treatment, was able to enhance significantly IL-1β and TNF-α, in D cells. Since the increase induced by GW6471 is significantly higher than the MPP^+^ group it is possible to suppose that the effect is cyclo-oxygenase-2 (COX-2)-dependent. In fact, previous reports have demonstrated that PPAR ligands modulate the LPS-stimulated synthesis of polyunsaturated fatty acid (PUFA) derivatives *via* the COX-dependent pathway and in particular, GW6471 is able to increase COX-2 protein expression (Chistyakov et al., 2020), which could be induced by MPP^+^ (Luchtman et al., 2013). Moreover, it is well established that IL-1β/TNF-a mediates the induction of COX-2 (Chen et al., 2000; Samad et al., 2001; Medeiros et al., 2010), which may in part explain our data.

In summary, our data have shown for the first time the protective role of OlGly in both not differentiated and differentiated MPP^+^-treated SH-SY5Y cells as an experimental model of neurotoxicity. The proposed mechanism is through PPARα and by modulating the levels of eCB and eCB-like mediators, possibly *via* FAAH inhibition. Other effects of OlGly have, in fact, been previously ascribed both to activation of PPARα and inhibition of FAAH (Rock et al., 2021). Moreover, the present study investigated the functional features of the eCB system systematically either in not differentiated and differentiated SH-SY5Y cells treated with MPP^+^, a piece of information that was missing in the literature.

## Conclusions

Our study clearly demonstrates that OlGly improves MPP^+^-induced neuronal damage by enhancing cell viability, improving neuroinflammation, and modulating the eCBome in SH-SY5Y cells. Notably, PPARα-mediated pathways mediate these protective effects. Therefore, though our study may suggest a protective role of OlGly in this cell-based model of neurotoxicity, further experiments using an adequate *in vivo* model of PD is mandatory to confirm that these benefits may be extended to the preclinical scenario in this disease.

## Data availability statement

The original contributions presented in this study are included in the article/Supplementary material, further inquiries can be directed to the corresponding author/s.

## Author contributions

FP and VD designed the research and wrote the manuscript. FP, AL, IC, RV, HK, CM, and FI conducted the research. FP and AL analyzed the data. All authors contributed to the article and approved the submitted version.
