# Supplementary material for: The endocannabinoidome mediator N-oleoylglycine is a novel protective agent against 1-methyl-4-phenyl-pyridinium-induced neurotoxicity
Source: Front Aging Neurosci. 2022 Oct 14;14:926634. doi: 10.3389/fnagi.2022.926634 (PMC9614236; doi:10.3389/fnagi.2022.926634)
Supplement: Supplementary file 1 [file Table_1.DOCX]

Supplementary Material

**Supplementary Table 1**: Primers sequences used in qPCR analysis

| ***Gene*** | ***FORWARD (5’->3’)*** | ***REVERSE (5’->3’)*** | ***Ref numb*** |
| --- | --- | --- | --- |
| **CB1** | TCTGTTCATCGTGTATGC | CTTGGCTAACCTAATGTCC | XM_006715330.1 |
| **CB2** | CGCTATCCACCTTCCTACAA | TAGTGCTGAGAGGACCCA | XM_005245737.2 |
| **NAPEPLD** | CGGAGCTTATGAACCGAGGTGG | GAGCCTTGACTCACCTTGCCGA | XM_006725056.1 |
| **FAAH** | GGCGGAGTGCGACAGCGTAG | GCCACTCTTGCTGAGGCGGT | NM_001441.2 |
| **DAGLα** | TCCTGGTGATCGAGTTCATCT | CATTCCGAGGGTGACATTCTTG | XM_005274230.2 |
| **DAGLβ** | TGGTGGATTGGCATTCTGACG | TTCCTCTCATGCTGACACACA | NM_001142936.1 |
| **MAGL** | AAACGAGGATCCGCTGCGCTC | ATGAGGGCCTTGGGTGTGCCT | NM_001003794.2 |
| **PPARα** | TTCGCAATCCATCGGCGAG | CCACAGGATAAGTCACCGAGG | XM_006724271.1 |
| **S16** | TCGGACGCAAGAAGACAGCGA | AGCAGCTTGTACTGTAGCGTG | XM_005259137.1 |
